# Comparative Pore Structure and Dynamics for Bacterial Microcompartment Shell Protein Assemblies in Sheets or Shells

**DOI:** 10.1021/acsomega.4c02406

**Published:** 2024-07-18

**Authors:** Saad Raza, Daipayan Sarkar, Leanne Jade G. Chan, Joshua Mae, Markus Sutter, Christopher J. Petzold, Cheryl A. Kerfeld, Corie Y. Ralston, Sayan Gupta, Josh V. Vermaas

**Affiliations:** † MSU-DOE Plant Research Laboratory, Michigan State University, East Lansing, Michigan 48824, United States; ‡ Biological Systems and Engineering Division, Lawrence Berkeley National Laboratory, Berkeley, California 94720, United States; § Molecular Biophysics and Integrated Bioimaging Division, Lawrence Berkeley National Laboratory, Berkeley, California 94720, United States; ∥ Department of Biochemistry and Molecular Biology, Michigan State University, East Lansing, Michigan 48824, United States; ⊥ Molecular Foundry Division, Lawrence Berkeley National Laboratory, Berkeley, California 94720, United States

## Abstract

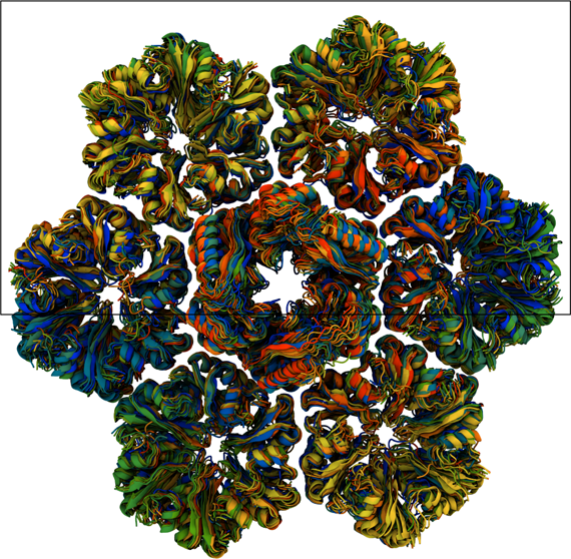

Bacterial microcompartments (BMCs) are protein-bound organelles found in some bacteria that encapsulate enzymes for enhanced catalytic activity. These compartments spatially sequester enzymes within semipermeable shell proteins, analogous to many membrane-bound organelles. The shell proteins assemble into multimeric tiles; hexamers, trimers, and pentamers, and these tiles self-assemble into larger assemblies with icosahedral symmetry. While icosahedral shells are the predominant form *in vivo*, the tiles can also form nanoscale cylinders or sheets. The individual multimeric tiles feature central pores that are key to regulating transport across the protein shell. Our primary interest is to quantify pore shape changes in response to alternative component morphologies at the nanoscale. We used molecular modeling tools to develop atomically detailed models for both planar sheets of tiles and curved structures representative of the complete shells found *in vivo*. Subsequently, these models were animated using classical molecular dynamics simulations. From the resulting trajectories, we analyzed the overall structural stability, water accessibility to individual residues, water residence time, and pore geometry for the hexameric and trimeric protein tiles from the *Haliangium ochraceum* model BMC shell. These exhaustive analyses suggest no substantial variation in pore structure or solvent accessibility between the flat and curved shell geometries. We additionally compare our analysis to hydroxyl radical footprinting data to serve as a check against our simulation results, highlighting specific residues where water molecules are bound for a long time. Although with little variation in morphology or water interaction, we propose that the planar and capsular morphology can be used interchangeably when studying permeability through BMC pores.

## Introduction

Bacterial microcompartments (BMC) are self-assembling protein-based organelles found in various bacteria.^1,2^ BMCs are thought to have evolved to facilitate catalysis in difficult or dangerous reactions. The semipermeable BMC shell protects the bacterial cytosol from the toxic effects of unstable or reactive intermediates that are present in these metabolic pathways by sequestering these intermediate products.^3^ Spatially confining these reaction pathways also increases their metabolic efficiency, in part by creating local high concentrations for enzyme substrates.^4^ Enzyme compartmentalization increases the rate of catabolism which increases fitness.^5^ BMC shells also serve to protect encapsulated enzymes from deleterious metabolites, such as O_2_ for oxygen-sensitive enzymes.^6,7^ For all of these reasons, BMC shells are emerging as engineering platforms for abiotic and biotic catalysis.^8−11^

A crucial limitation for engineering new catalytic pathways into BMC shells is whether reactants and products permeate across the shell. Natural BMCs are permeable to a wide range of metabolites, such as bicarbonate and Calvin-Benson-Bassham cycle intermediates to facilitate carbon fixation in cyanobacterial carboxysomes,^12^ or reactants and products for propanediol,^13^ ethanolamine,^14^ and fucose or rhamnose^15^ catabolic pathways. It is thought that the permeation occurs through the central pores within the individual tiles identified from molecular structures,^16,17^ corroborated by molecular simulation for metabolites through these pores.^18−20^

**Figure 1:**
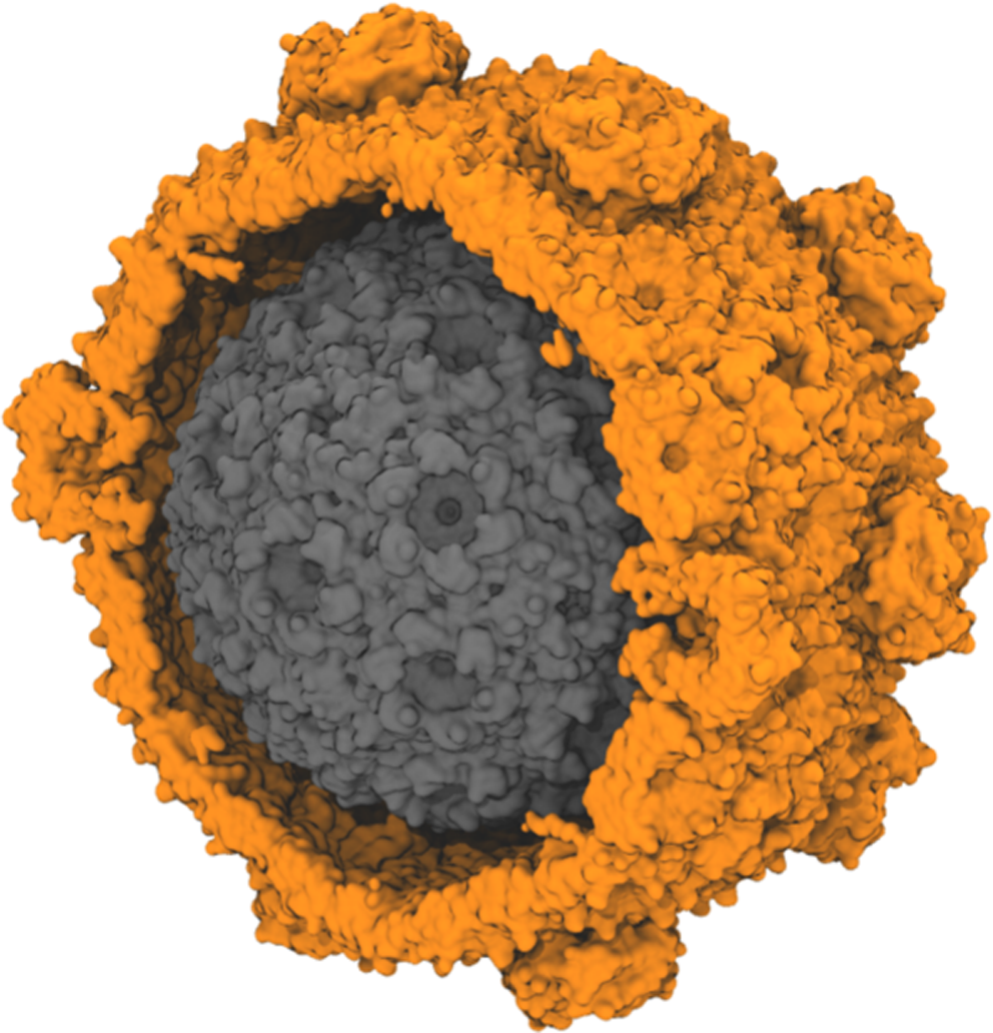
Size comparison for different BMC shell architectures made from *Haliangium ochraceum* shell proteins. The shown structures are determined by cryo-EM, and represent a minimal shell (gray, PDB: 6OWG, approximately 2.4 M atoms when solvated for simulation)^21^ and a full HO shell (orange, PDB: 6MZX, approximately 10 M atoms when solvated for simulation).^22^ The minimal shell contains only hexameric and pentameric units, while a typical full shell also includes trimeric proteins, some of which form double stacks in the shell. The diameter of the minimal model BMC is around 22 nm, while the full shell diameter is approximately 40 nm.

Molecular simulations can model the permeation for any metabolite across these pores explicitly. A common approach is to use isolated protein tiles in solution as a model for the BMC shell, effectively measuring permeability in the dilute limit.^19,20^ More recently, intact shells have been simulated.^18^ While intact shells are a more accurate representation for the molecular nanostructure, simulating these assemblies at the atomic scale substantially raises the cost of determining molecular permeability. The increased computational cost is particularly acute when trimeric shell components are included, as the shell size increases substantially (Figure 1). Since most of the simulation volume for these intact shells is water, an alternative intermediate system that reflects the symmetry of BMC shell components would be ideal to better balance computational cost and accuracy.

To explore this idea, we leverage the fact that BMC shell proteins do not always form shells. Alternative morphologies such as sheets of hexamers^23^ or arrays of trimeric BMC proteins^24^ have been experimentally characterized. Cylinders have been reported *in vitro*(25) after modifying the ratio of shell components for the *Haliangium ochraceum* (HO) shell.^26−28^ While the HO shell is quite small, other BMCs, such as carboxysomes, form large icosahedral structures with large triangular planar facets connected together with vertices.^29^ The vertices for the icosahedron are filled by pentameric shell proteins, but the approximately planar facets are thought to be a mixture of hexameric and trimeric proteins analogous to the hexameric and trimeric proteins in HO.^1,30^ In this study, we design a theoretical and computationally feasible mode of the BMC shell protein in sheet conformation and we compare the dynamics and structure of a small periodic BMC sheet model developed *in silico* to an equivalent shell fragment derived directly from experimental structures.^22^ Crucially, we find that the dynamics and pore diameter for planar or shell structures are similar, indicating that the permeability for the developed sheet system is similar to the permeability that would be expected *in vivo*.

## Methods

### Structure Preparation

**Figure 2:**
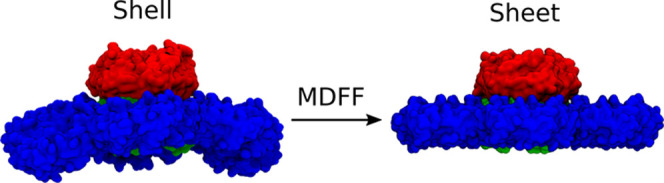
Using MDFF methods, we convert the shell-like curved facet of BMC (left) from a reduced PDB ID:6N0F to a sheet-like conformation (right). Hexamer tiles are in blue, and the stacked trimers are a dimer of two trimers (one red, one green), resulting in the red trimer protruding from the plane of the shell facet, in which the green trimer is embedded.

Fundamentally, there are two different models prepared in this study: a curved facet from a larger BMC shell and a planar arrangement of the same proteins. The curved shell facet (labeled as shell in Figure 2) starts from existing structures determined from cryo-EM, specifically the 6N0F structure that features a single stacked BMC trimer surrounded by BMC hexamers^22^ with a closed BMC trimer. The three pentameric units at the edges of the 6N0F structure were removed from the structure prior to the simulation. The six hexamer tiles around a central trimer allow for substantial sampling for six pores simultaneously in the same simulation system. The starting structure is prepared in VMD^31^ using the solvate and autoionize plugins to create a 234 × 203 × 234 Å^3^ system suitable for further simulation.

To create a planar sheet-like structure, we use molecular dynamics flexible fitting (MDFF)^32,33^ to flatten the initial structure (Figure 2). Since MDFF requires an electron density, either real or synthetic, as the target, we generated a nominally flat target conformation for the hexamers. Starting with the curved facet, we first determine a vector normal to the trimer pore by creating vectors  and  from adjacent protein pairs in the upper trimer. The cross-product of these two vectors is normal to the trimer pore and can be brought to a specific axis using the transvecinv routine in VMD.^31^ The trimer is moved to the origin, and this procedure is repeated for each hexamer individually to create the sheet arrangement. After transformation, the synthetic target density map was generated at 4 Å resolution via the mdff plugin within VMD,^31^ using the combined atomic model of the flattened hexamers. The Tcl scripts for generating the flattened hexamers using rigid body transformation and generating the synthetic density map of the flattened system are available via Zenodo.^34^

**Figure 3:**
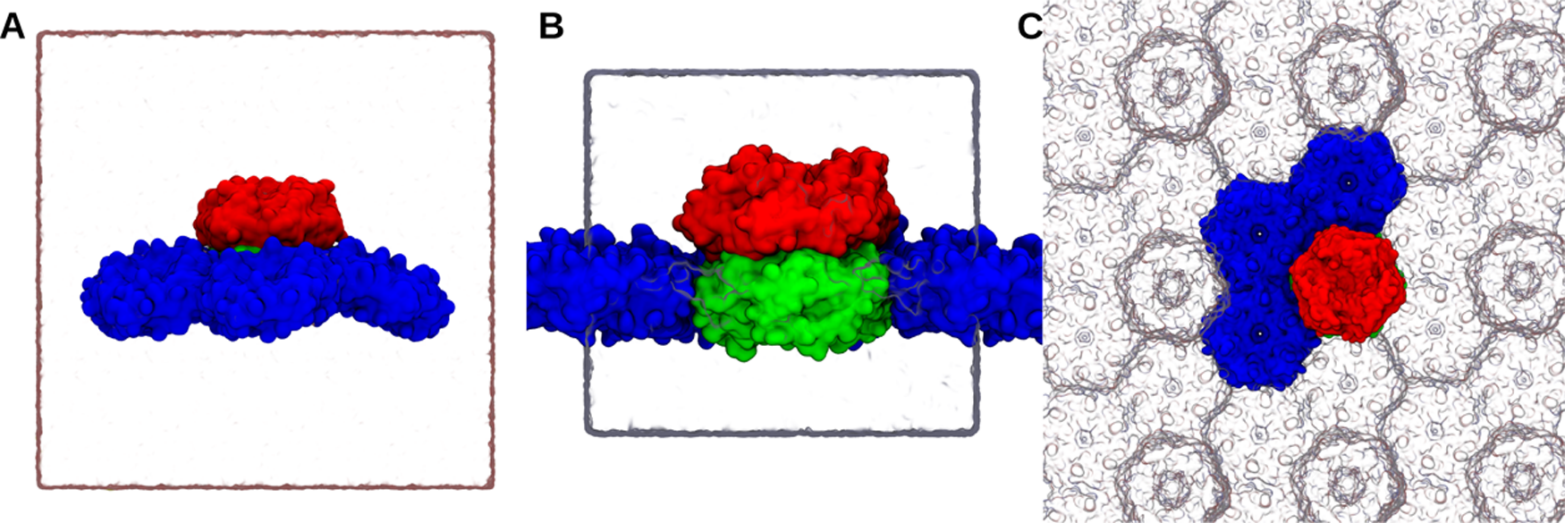
Simulation box of curved shell (A) and planar sheet conformation (B) with water box represented as the white rectangular which also represent the periodic boundary of the system. (C) Top view of sheet simulation system comprising three hexameric units and one trimer dimer which can mimic the planar sheet conformation and keeps the system periodic in a rectangular simulation box. Periodic images of the facets are presented as glass bubble. Hexamers are represented as blue surface representation, trimer upper half as red, and lower half as green surface representation. Planar conformation can be seen mimicking a lipid membrane where the hexameric units are touching the periodic trimeric subunits. Animations for these simulation systems are available as Animations S1 and S2.

In order to create an effectively infinite planar sheet of BMC shell proteins, the flattened facet was arranged in the *X*–*Y* plane such that the pore normal is aligned with the *Z*-axis. Current molecular dynamics (MD) simulation algorithms, particularly the GPU-resident integrator of NAMD,^35^ are most performant on simulation boxes with orthogonal box dimensions. We reduce the system size and maintain an orthogonal periodic boundary condition by packing three hexamer and one trimer tile into a rectangular unit cell such that the trimer tile is always surrounded by hexamer tiles as is the case in cryo-EM structure.^22^ The *X*–*Y* plane dimensions were set based on the distance between repeating units within the structure. Once optimized in this manner and solvated, the unit cell dimensions for the final structure in Figure 3B are 130 Å × 170 Å × 152 Å. Figure 3C places the repeating unit within the larger context, while Figure S1 explicitly shows a surface representation for the repeating unit and how it tiles together.

### Simulation Protocol

Classical molecular dynamics simulations were carried out using the CHARMM36m protein force field^36^ in explicit TIP3 solvent^37^ using NAMD.^35^ Minimization and a brief equilibration were performed with NAMD 2.14 prior to unbiased production simulations using the GPU-resident integrator on NAMD 3.0a9 to maximize performance.^35^

Most simulation parameters were shared when running the shell and sheet-like structures shown in Figure 3. Temperature was controlled by using the Langevin thermostat at 298 K with 1 ps^–1^ damping. Hydrogen bonds were handled with SETTLE algorithm to enable 2 fs timesteps.^38^ Long-range nonbonded Lennard-Jones (LJ) cutoff was set to 12 Å. Long-range electrostatic interactions were calculated with particle mesh Ewald (PME) grid with 1.2 Å spacing.^39,40^ The switching between nonbonded interaction and electrostatic is done after 10 Å. LJ correction is applied to improve energy conservation during switching.^41^ Energy minimization of the system was initially performed using the 1000 steps of conjugate gradient in NAMD.^42^ Both systems were briefly equilibrated for 50 ps in the NPT ensemble using 5 Å margin to allow the box to adjust after any distortion from minimization prior to transitioning to the GPU-resident integrator. Simulations in the production NPT ensemble were run for 1 μs for shell and 1.5 μs for sheet structures shown in Figure 3.

The difference between the sheet and shell structures is in the pressure control. A Langevin barostat was used to maintain pressure at 1 atm,^43^ with the shell fragment using isotropic pressure control and the sheet using anisotropic pressure control. For sheet simulations, the x- and y-dimensions are tied to the expansion and contraction of the individual shell proteins, analogous to a lipid bilayer. Unlike lipid bilayer systems, where the membrane plane would be uncoupled except through the barostat, the protein itself can grow and shrink in different amounts along the axes parallel to the sheet surface. Thus, shell fragment simulations used a flexible simulation box with a fixed aspect ratio, while production simulations for the sheet structure applied anisotropic pressure control that varied independently in every dimension.

### X-ray Footprinting with Mass Spectrometry (XFMS)

Two samples were prepared for X-ray Footprinting with Mass Spectrometry (XFMS). The first sample was taken from intact synthetic HO shells, assembled following the steps laid out in prior literature.^44^ The second sample was the purified component HO BMC-H protein (hexamer tile), which spontaneously forms uniformly oriented sheets at high concentrations;^28,44^ this was diluted to the extent that no sheets formed.

Samples were exposed at the Advanced Light Source beamline 5.3.1 with exposures of 0, 100, 250, 500, 750, 1000, and 2000 s, using a horizontal capillary as previously described.^45^ Post exposure, the samples were digested using trypsin enzyme (Promega) overnight at 37 °C at pH 8 in 50 mM ammonium bicarbonate buffer. Liquid chromatography–mass spectrometry (LCMS) was conducted on an Agilent 6550 iFunnel Q-TOF mass spectrometer (Agilent Technologies, Santa Clara, CA) coupled to an Agilent 1290 LC system (Agilent). Peptide samples were loaded onto a Sigma-Aldrich Ascentis Peptides ES-C18 column (2.1 mm × 100 mm, 2.7 μm particle size; Sigma-Aldrich, St. Louis, MO) via an Infinity Autosampler (Agilent) with Buffer A (2% Acetonitrile, 0.1% Formic Acid) with flow rate 0.400 mL/min. Peptides were eluted into the mass spectrometer via a gradient with an initial condition of 5% buffer B (98% acetonitrile, 0.1% formic acid) increasing to 90% B over 15 min. The data were acquired with MassHunter B.05.00 operating in Auto MS/MS mode whereby the three most intense ions (charge states 2–5) within *m*/*z* 300–1400 mass range above a threshold of 1000 counts were selected for MS/MS analysis. MS/MS spectra were collected with the quadrupole set to a narrow resolution and collision energy to optimize fragmentation. MS/MS spectra were scanned from *m*/*z* 100 to 1700 and were collected until 40000 total counts were collected or for a maximum accumulation time of 333 ms. Parent ions were excluded for 0.1 min following MS/MS acquisition.

XFMS peptide identification and analysis was performed using the Byos (Protein Metrics, Inc.) integrated software platform at the Molecular Foundry as previously described.^46^ Briefly, the abundances of the identified unmodified and modified peptides at each irradiation time point area were measured from their respective extracted ion chromatogram of the mass spectrometry data collected in the precursor ion mode. The fraction unmodified for each peptide was calculated as the ratio of the integrated peak area of the unmodified peptide to the sum of the integrated peak areas from the modified and unmodified peptides. The dose–response curves (fraction unmodified vs X-ray exposure) were fitted to single-exponential functions, producing a *k*-value (s^–1^). The ratio of k-values provided the relative change in solvent accessibility between the sheet and shell forms.

### Solvent Accessibility Analysis

Structure files and MD simulation trajectories were visualized and analyzed using Python-enabled VMD 1.9.4a58.^31^ Python-enabled VMD provides an interface to apply the numpy numerical library^47^ and plotting tools like matplotlib.^48^ System stability was assessed first by computing the root-mean-square deviation (RMSD) for the entire trajectory. Through RMSD analysis, we determined the equilibration period for each simulation system. The RMSD for the shell fragment stabilized in approximately 200 ns, while that for the sheet stabilized more slowly. For subsequent analysis, only the last 800 ns for shell and 1000 ns of sheet configuration were used. The fluctuation of each atom in the hexamer and trimer monomers was calculated using root-mean-square fluctuation (RMSF). The solvent-accessible surface area (SASA) was computed residue-wise, accelerated by a modified analysis routine that has been committed upstream to the VMD developers. Water contacts with the BMC hexamers and trimer were calculated using the contact function in VMD to track the number of unique water molecules within 5 Å of a given residue. Beyond computing water contacts, we also calculated the water retention time around the BMC hexamers and trimers. The residence time was determined by tracking frame by frame if a water molecule was initially within 5 Å of a given residue and stopping the clock when the water molecule was further than 8 Å away from the residue.

To compare directly with experimental observations based on hydroxyl radical footprinting data, we subdivided the shell fragment hexamers based on their water accessibility. For the purposes of comparison with intact shells, we evaluate hexamer monomers within the shell fragment system that are interfacing directly with the trimer tile (red in Figure S2). When compared to dilute hexameric tiles in solution, we compare with hexamer monomers in the shell fragment system that are solvent-exposed (blue in Figure S2). This facilitates a direct comparison with the companion experiment.

### Pore Analysis

The primary analysis of interest is determining the pore size within a BMC protein tile. Borrowing from membrane protein studies, we used the HOLE program^49^ to determine pore radius along the channel formed at the center of BMC hexamer and trimers tiles. The HOLE algorithm works by finding a maximum sphere fitting inside the cavities of protein along the *z* axis of the protein. The HOLE program was written to analyze a single conformation, so for full trajectory analysis, an additional wrapper is required. While other tools such as MDAnalysis have such wrappers already built-in,^50,51^ optional parameters were essential to guiding HOLE along the pore of interest. In this vein, we wrote HoleHelper, a Tcl plugin to VMD that facilitates using HOLE for our specific systems with the VMD atomselection language. HoleHelper is available on github for public download and use (https://github.com/joshua-mae/HoleHelper).^52^ The simulation snapshots that were judged to be equilibrated by RMSD were used to determine the pore size probability distribution.

## Results

Molecular simulation provides a unique perspective to address specific mechanical and structural questions at the nanoscale and has been called a ”computational microscope”.^53,54^ Turning this microscope to BMC shell protein assemblies, the key question is whether the pores respond at all to the environment, similar to the opening and closing mechanism for mechanosensitive channels.^55^ By also checking for stability and comparing our structures to experimental observables, we are confident that the BMC shell components are closer in nature to aquaporins and do not uniquely depend on external pressure or their environment to govern pore dynamics.

### Structural Stability Considerations

**Figure 4:**
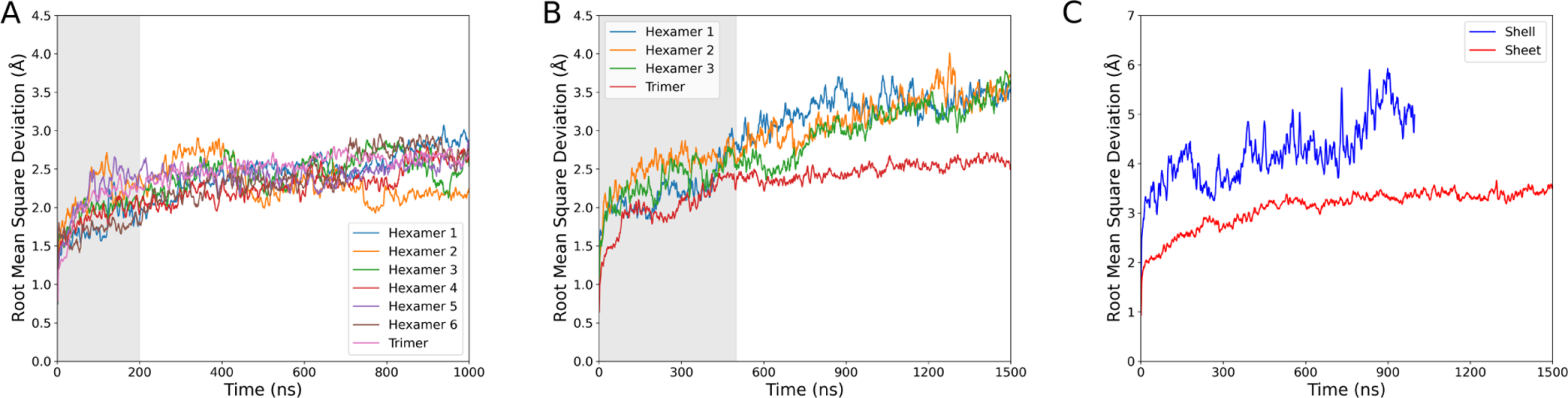
Root mean square deviation (RMSD) of carbon α for each hexamer and trimer when taken from the (A) shell fragment or (B) sheet simulation systems from Figure 3. (C) RMSD of the entire facet in the shell and sheet conformation. The reference structure to align to when assessing the RMSD was a tile from the initial 6N07 structure,^22^ and is plotted individually for each individual tile within the system. RMSD has been smoothed with a rolling window average over 5 consecutive frames. The shaded gray area is the simulation equilibration time set for the shell (200 ns) and sheet (500 ns) conformations.

Prior to any pore geometry comparison, we use the root-mean-square deviation (RMSD) over time to assess general protein stability within our simulation environment. Since the resolution for the original cryo-EM structure is 3.6 Å,^22^ we anticipate an RMSD similar in magnitude to this resolution, as this relationship has been noted previously for membrane proteins.^56^ That is indeed what we see in Figure 4A,B, with extended simulation only yielding RMSDs that occasionally exceed the solved structure resolution. The shell fragment routinely has lower RMSD than the sheet, suggesting that there are subtle structural changes that have occurred, as the reference structure for each tile is identical between both states. Since the RMSD change is so small, and largely confined to the hexamers, the overall secondary structure is consistent between states.

When computing the RMSD for the entire system, rather than of an individual tile within the assembly, the trend is reversed. The RMSD of the protein components for the full system is higher in the shell fragment as individual subunits move relative to one another during the simulation (Figure 4C). While the larger number of protein units within the shell fragment undoubtedly increase the RMSD somewhat, the overall conclusion that the structure is consistent across the simulation is unchanged.

**Figure 5:**
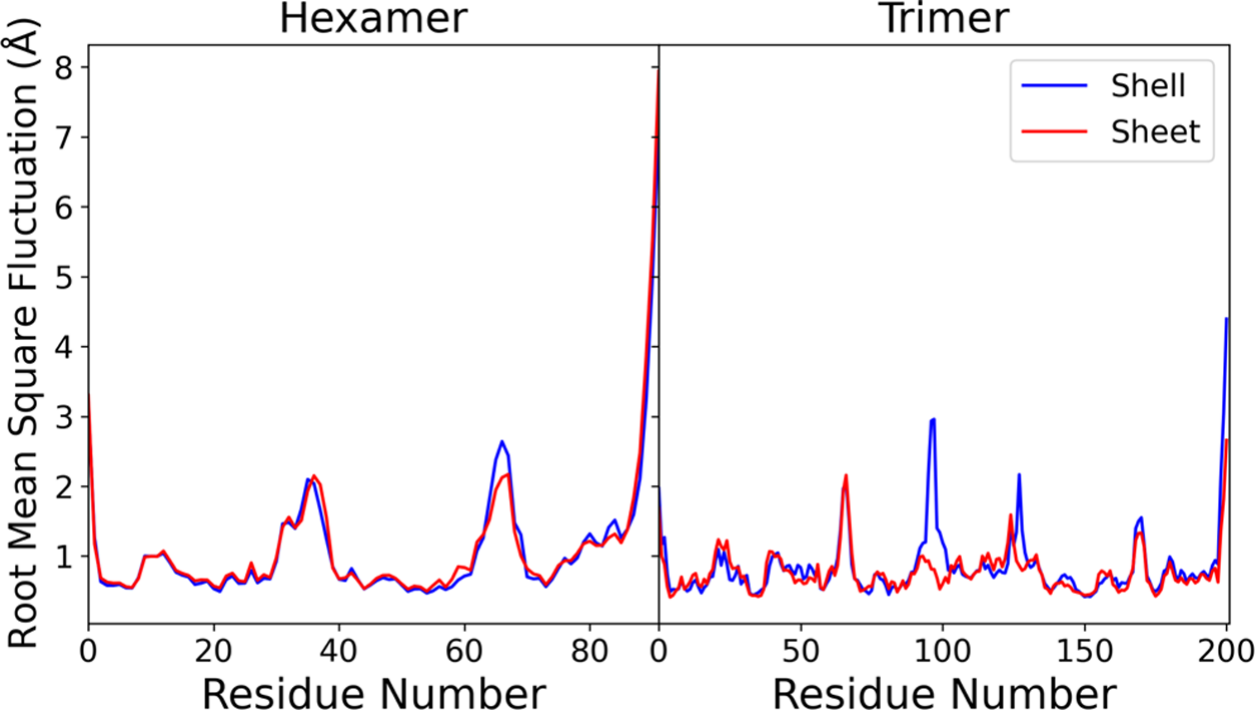
Root mean square fluctuation (RMSF) of C_α_ for each hexamer and trimer monomer when taken from the shell fragment and sheet simulation systems from Figure 3. The reference structure for alignment when assessing the RMSF was the initial simulation frame.

We identified from RMSD analysis that there is still a slight upward trend in the RMSD values in sheet conformation just after simulation begins. Thus, when we calculate equilibrium properties, we do so only on the time points after 200 ns in the shell and after 500 ns in the sheet when we judge the structure to be equilibrated (Figure 4). When evaluating the RMSF on these states (Figure 5), we see only slight changes on a per-residue basis, suggesting that the dynamics are similar in both states. The largest RMSF is observed at the N and C terminal loops within the hexamer monomers. These are adjacent to regions that are not resolved by cryo-EM, and may just be naturally flexible and have high RMSF in general. The largest changes in the RMSF are at residues 66–70 in the hexamer, which is a connecting loop between the secondary structure elements. Similarly in the trimer, the high fluctuation regions in the interior are also connecting loops between structural elements far from the central pore. The larger variation between conditions in the trimer shown in Figure 5 likely results from reduced sampling, as there are only three monomers in each system compared with many more copies of the hexamer monomer.

It is worth noting at this point that while individual proteins are reasonably intact, we also observed transient gap openings in our simulation within the assembled sheets. These gaps are large enough to let water and gases pass through the interfaces but likely still hinder the product and reactant movement through these transient channels. Thus, larger metabolites would likely be confined to the standard pores within the BMC. Although we do not see any opening in shell conformation from our simulation trajectory, prior simulations have observed water and gas permeation through transient interfaces in a complete carboxysome shell,^18^ and thus this is not unexpected. We anticipate that the curvature imposed as the shell fragment was flattened into a sheet may have loosened the tight packing that typically characterizes a HO shell.

### Pore Dynamics in BMC Shell Fragments and Sheets

**Figure 6:**
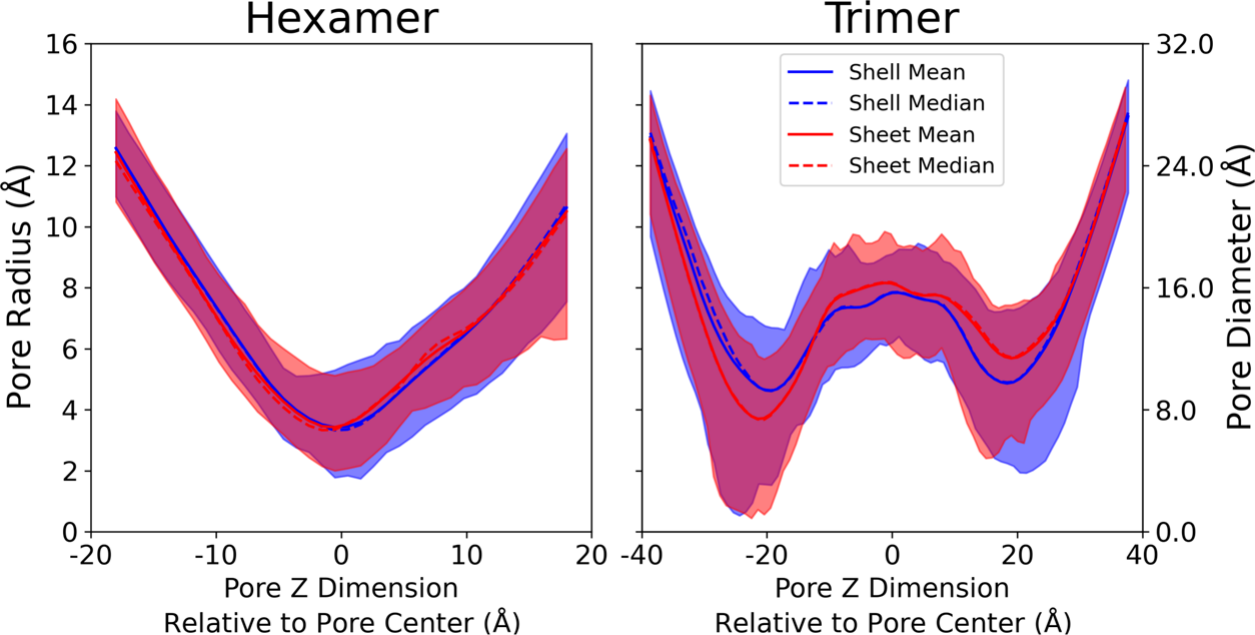
Distribution of pore radii along the central pore for hexameric and trimeric protein tiles in either a shell fragment or sheet morphology. In this representation, the midpoint *Z* is at the geometric center of the hexamers or trimers that make up the pore, which is not where the bottlenecks occur. The shaded area represents the maximum and minimum distribution of pore sizes observed during the simulation. The solid line and dashed line represent the average pore size and median pore size, respectively, observed in the simulation trajectory, across all 6 hexamer tiles (shell fragment) or 3 hexamer tiles (sheet).

The small variations in RMSD from Figure 4 leave open the possibility that the individual pores may change their structure when shell proteins are exposed to different local environments. Since pore size and dynamics can alter the metabolite transport, monitoring pore fluctuations over time is essential. On average, we find that the pore radii, both in their ranges and their average, are highly consistent between simulations run in either condition (Figure 6). On average, the hexamer tile has a central pore with a bottleneck diameter of 6.9 Å in the shell or 7.1 Å in the sheet, with a distribution whose mean is approximately equal to the median. This minimal change indicates that the hexamer pore is invariant to the local protein environment and exhibits similar variation in size across the simulations. The relative distribution also has a similar pattern and no large changes are seen in the shell and sheet conformations (Figure S3).

The double-stacked trimer pores exhibit substantially greater variation in the range of possible pore diameters at the bottleneck, at ±20 Å. In some conformations, particularly at the beginning of the simulation where the structure has not diverged very far from the closed starting point created by the 6N07 starting structure, the trimeric pore is effectively closed at the bottleneck. This closed pore would likely represent a large barrier to permeation of all but the smallest molecules. However, in other conformations, the trimeric pore has a substantially larger diameter. On average, the trimers expose a larger pore for metabolites to transit across. Thus, we anticipate that the trimer may be the preferred path for some molecules to permeate that cannot be accommodated by the smaller hexamer.

For small-molecule permeation, pore dynamics are essential. As the starting structure is taken from a closed starting structure, our initial structures exhibit a closed conformation for the hexamer and trimer complexes. The minimum hexamer opening often occurs at time zero, starting with a bottleneck radius of only 2 Å (Animations S1–S4). The bottleneck expands quickly as the pore hydrates and side chains rearrange to a typical radius of 5–6 Å (Figure 6). The trimeric complex exhibits even stronger dynamics, with an effectively closed pore in the initial structure.^22^ The HOLE output highlights an expanding pore over time (Animations S3 and S4).

From the starting structure, the amino acid side chains that line the pore effectively close the pore and would likely bar many small molecules from transiting the BMC shell. However, during simulation, these pore-lining residues can readily move without large secondary structure variations to a more open state, as shown in Figure S4. In our simulations, we find that once the pore is opened, the pore will not dehydrate and close again for a long time during the simulation, although side chains may sporadically occlude the pore. These briefly closed conformations are visible in the supporting animations and have included a still image of one such closed conformation as Figure S5. Shell and sheet assemblies open up in a similar manner, and both systems demonstrate sporadic pore closure. While classical simulation water models are unusual in many respects, we anticipate that in this case the pore structure observed in cryoEM was more a result of the low temperature at which the samples were collected. It is possible that the pore may reclose fully if the simulation were to be extended; however, given how few trimers we have in our simulation systems, the cost in computer time to observe reclosing events was thought to be prohibitive to depend on stochastic sampling alone to reclose the pore.

From the pore diameter distribution, we note an asymmetric distribution in the top and bottom halves of the trimer (Figure S3). BMC trimers are thought to have a gating role,^2,22,24,30,57^ and so some asymmetry can be readily expected. While we are limited in our sampling for the trimer, with only a single copy in both scenarios, the observed asymmetry in Figure S3 may be real, driven by the need to gate substrate translocation.

Beyond pore dynamics, the sheet simulations in particular show small gaps that appear between tiles and these gaps are substantially larger than what is seen in the shell simulations (Animations S1 and S2). In our own internal testing, we could not identify barostat conditions that would disallow these gaps to form, suggesting that either the sheet is not packed perfectly during the flattening process or these sporadic openings are real when the highly curved HO shell is flattened. In model carboxysomes, water has been observed to transit the shell through the interface made between the edges of BMC hexamers.^18^ In our view, this suggests that these small gaps are also present in native HO shells, perhaps compounded by the flattening process to which we subjected them here.

### Water Interaction Analysis

**Figure 7:**
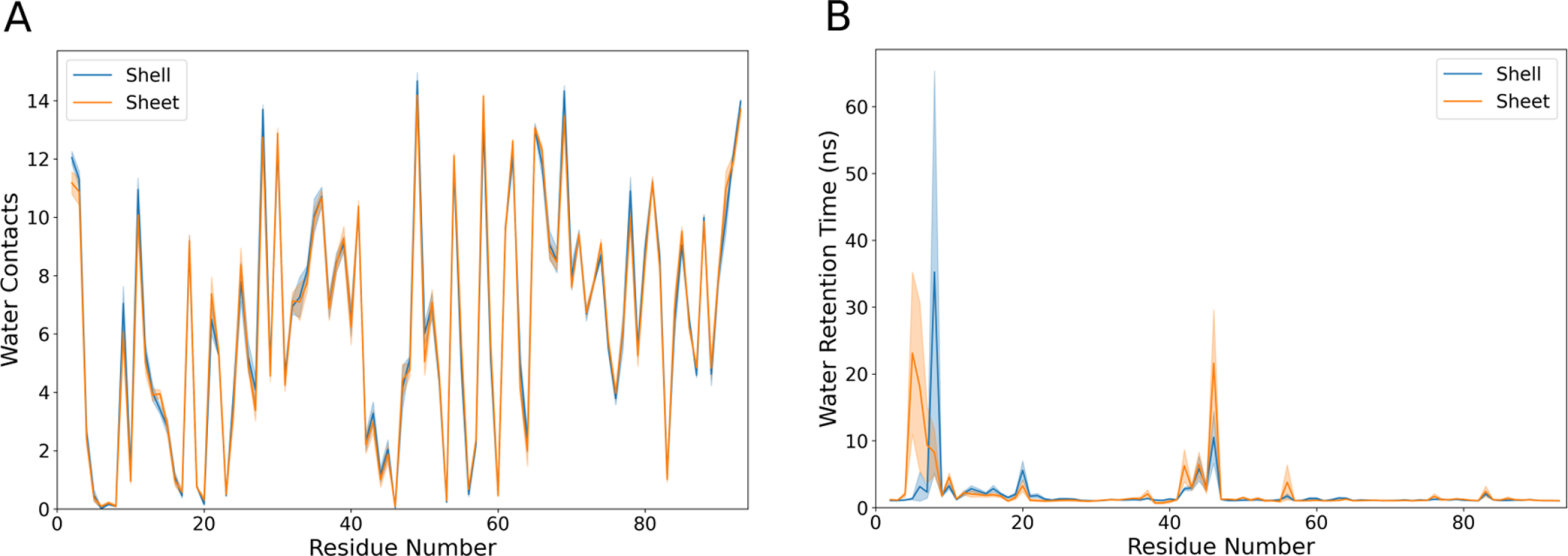
Hexamer tile interactions with water. (A) Quantification of the water contacts on a per-residue basis, counting the average number of pairwise contacts between amino acid residue atoms and water atoms that are under 5 Å in separation over the total trajectory. To maintain consistency with the semi-infinite sheet, the shell values reported here are averaged only over the three hexamer monomers nearest to the central trimer. (B) Measurement of the water retention times, based on how long on average a given water molecule remains within 5 Å of a given residue. The shaded area is the standard error of the mean for SASA and water retention for each residue in a hexamer monomer. Note that both quantities within the hexamer are averaged over the 18 monomers that are not solvent-exposed for the shell fragment system, and over all 18 monomers in the sheet system.

Beyond the proteins themselves, our simulation systems feature water to fill in the rest of the simulation volume. While the pore dynamics are likely most critical to permeability, observing changes in water interactions may be another avenue by which we can tease apart the subtleties of structural differences between sheet-like and shell-like structures. The per-residue water contacts vary minimally between the two tested conformations for the hexamer (Figure 7A), suggesting that even structural details are largely conserved at a global scale between the curved shell fragments and a larger sheet. This total picture of conserved contacts is retained if we expand our view to include the trimer. By mapping water contacts onto the structure, as in Figure S6, we visually see the same water contact patterning across both structures. Contacts are naturally highest on the protein periphery that are solvent-exposed, with the residues at the central bottleneck having roughly half the number of water contacts due to protein occluding many potential water interaction sites. In general, this suggests that both structures are facing the same solvent environment regardless of the exact geometry at play.

**Figure 8:**
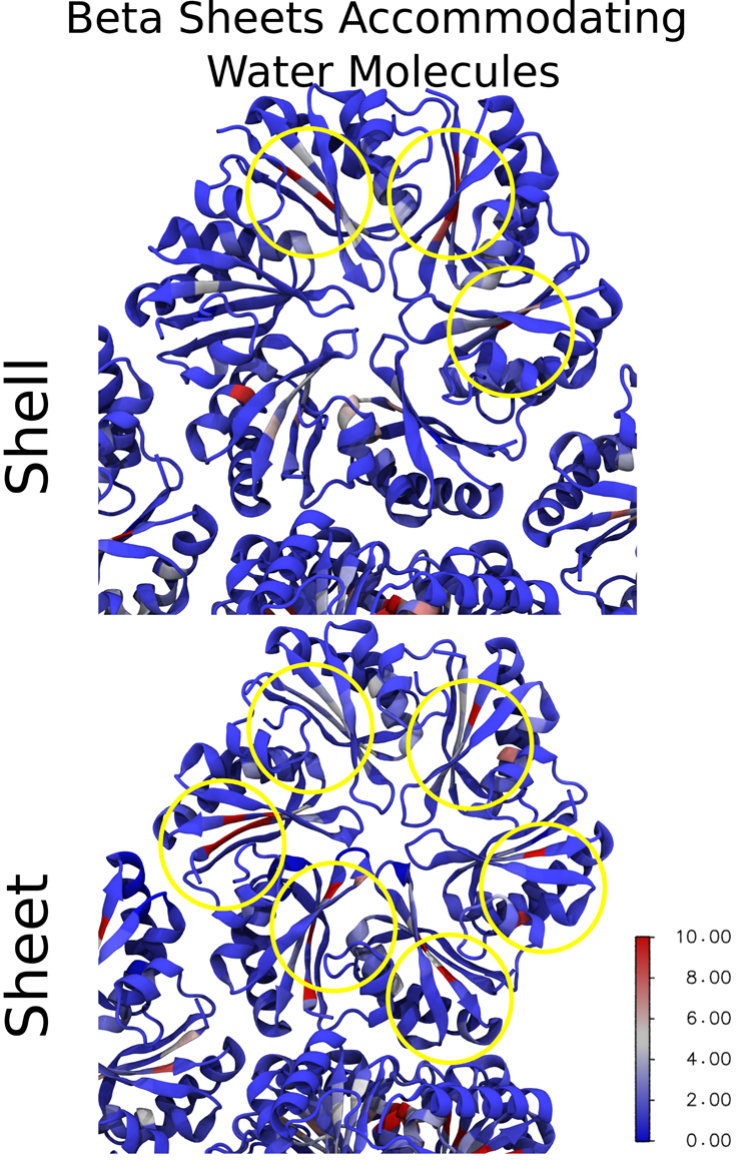
Water retention mapped to each residue on the hexamers and trimers in shell-like and sheet morphologies. Proteins are drawn in a cartoon representation where each residue is color-coded on the blue-white-red spectrum to represent the water retention time. The color bar measures the water retention time in nanoseconds.

**Figure 9:**
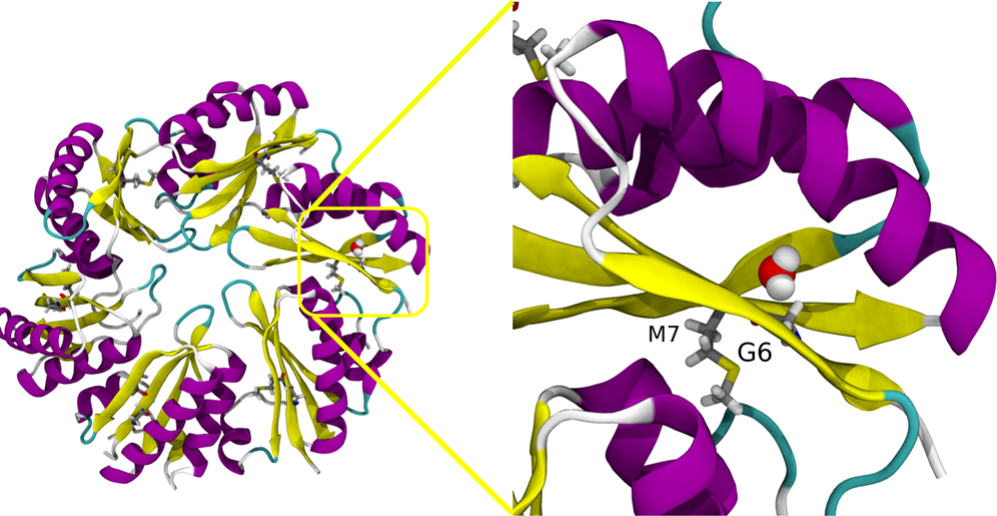
Retention of water in a pocket around the β sheet in hexamers near residues G6 and M7. Proteins are represented in cartoon representation, residues G6 and M7 in licorice, and water in VDW. The left panel represents the whole hexamer unit, and the right panel represents the water pocket.

Quantifying contacts alone is only one metric of interest. With an eye toward comparing to hydroxy radical footprinting data, where the timespan individual water molecules spend near specific protein residues is of interest, we quantify the residence time for water molecules near individual residues (Figure 7B). We see some increased retention of water molecules near specific residues, with substantial differences in the range of residues 5–11 (LGMIEVR), 20 (A), 41–48 (YVTAVRGD), 50–52 (VAA) and 83 (V) (Figure 7B). The strongest difference in water retention between sheets and shells occurs around G6, where water in the sheet conformation is retained for over 30 ns on average. This residue is on the border of an interstitial water site near β sheets within an individual hexamer (Figures 8 and 9). The residues lining the pores have much shorter water interactions, as water at the interface readily exchanges with the bulk.

### Correlating Simulation to XFMS

Our *in silico* work so far has emphasized that the differences between water access and pore formation are really minimal. Is it possible to use an experimental measure like XFMS, where amino acid hydroxylation induced by water ionization through X-ray exposure can be measured by mass spectrometry,^58,59^ to provide experimental support for these findings? Data for the hexamer in solution or as part of a shell are provided in Table 1. The *k*-values measure the hydroxyl replacement rate at specific residues where hydroxylation is possible. The replacement rate in solution can be greatly accelerated when compared to the complete shell, such as at the M7 residue, which has a very high ratio of hydroxylation rate in solution compared to when it is in the shell. This indicated that the M7 residue has a substantially lower availability to water when it is in the shell compared with the solution state. We did not directly simulate an isolated hexameric tile in solution, and not all hexamer monomers within our curved shell fragment are a good facsimile for the environment of a complete shell. Thus, we segment our data into two populations based on hexamer identity (Figure S2) to establish comparisons with Table 1. After this segmentation process, we made comparisons with structural metrics.

**Table 1:** XFMS *k*-Values at Different Positions on the BMC Hexamer for a Single Hexamer Tile in Solution and as Part of a BMC Shell Assembly^a^

residues	solution *k*-value	shell *k*-value	ratio
M7	(242.0 ± 9.6) × 10^–6^	(29.8 ± 8.2) × 10^–6^	8.12
M16, M23	(65.2 ± 2.1) × 10^–6^	(51.2 ± 1.4) × 10^–6^	1.27
Y34	(14.7 ± 1.9) × 10^–6^	(15.50 ± 0.71) × 10^–6^	0.95
Y41	(6.0 ± 1.7) × 10^–6^	(3.90 ± 0.55) × 10^–6^	1.53
K54	(14.00 ± 0.84) × 10^–6^	(6.50 ± 0.31) × 10^–6^	2.16
P77, P79, P88	(477.0 ± 9.1) × 10^–6^	(187.0 ± 8.9) × 10^–6^	2.54

a The ratio of these two rates tells us something about how water accessibility changes based on BMC protein morphology.

**Figure 10:**
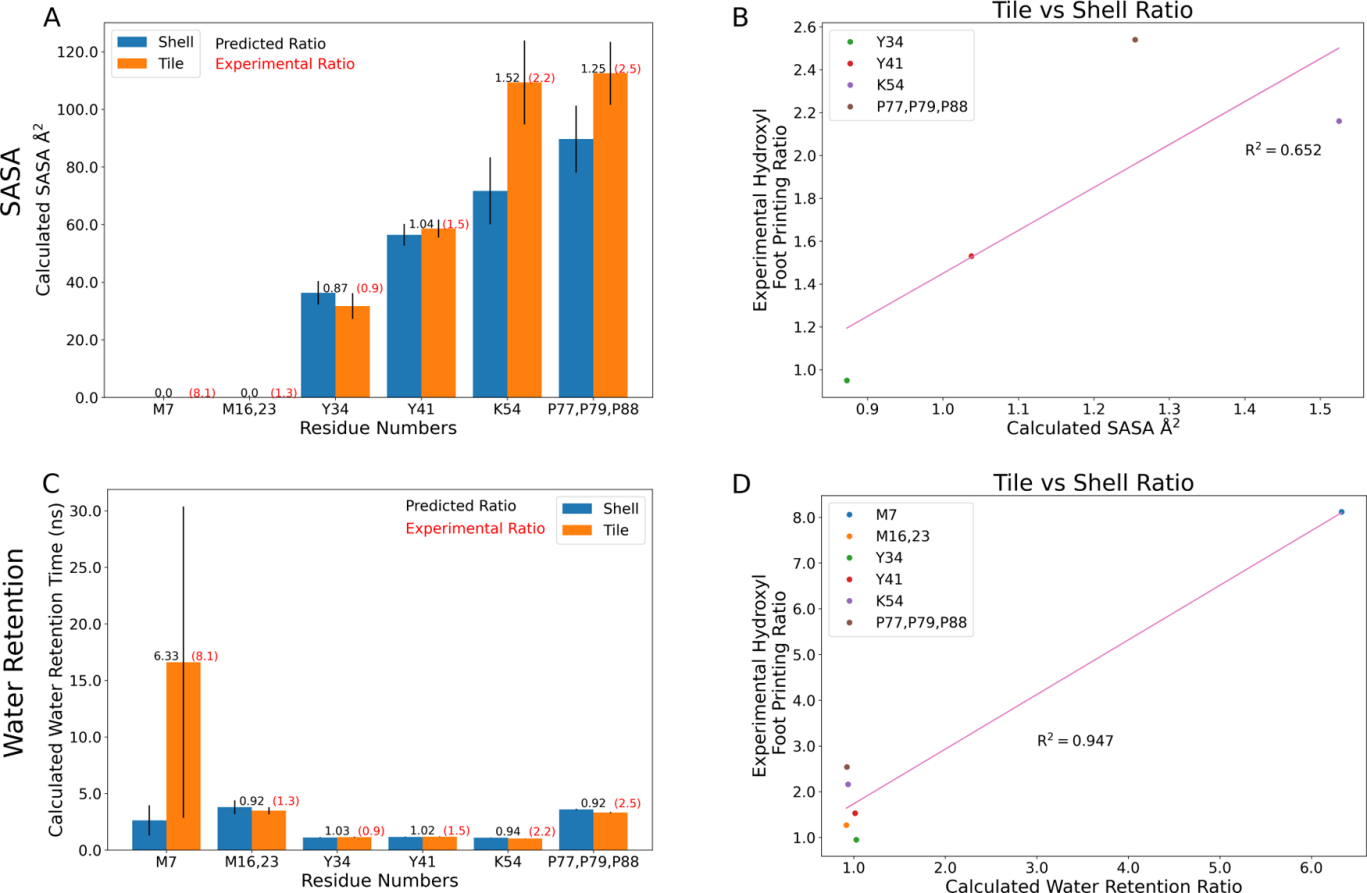
Measured solvent-accessible surface area (SASA) and water retention times across the residues where we have XFMS comparison data (Table 1), directly comparing the equivalent residues within our models that belong to exposed or buried monomers (Figure S2). The ratios of these quantities determined from simulation are reported in black above the histograms for (A) SASA or (C) water retention times, while the experimental equivalents are written in red. The error bar represents the standard error of the mean in SASA and water retention for the residues. The scatter plot comparing the reaction rate ratios to the ratio of (B) SASA or (D) water retention times has a line of best fit along with a correlation coefficient given in pink. For SASA correlation measures, residues M7 and M16,23 are not shown, as they have zero SASA values and thus a poorly defined ratio.

The solvent-accessible surface area (SASA) for individual residues has been previously demonstrated to correlate somewhat with hydroxyl radical footprinting data derived from XFMS.^60^ There certainly is a trend when evaluating the SASA overall for specific residues (Figure 10A,B). The fit improves if M7 is excluded from consideration, which might be reasonable as our SASA determination algorithm cannot find a water-accessible surface near this buried residue. However, from Figures 7 and 8, we know that water can access these buried residues near the β-sheet. Moreover, if water accesses these residues, then they may be present near these amino acids for a considerable duration (Figure 7B). Thus, the initial outlier of M7 may be explained by tracking how long a water molecule is present as this ratio can change substantially for buried residues (Figure 10C). Indeed, taking the ratio yields a very strong correlation coefficient (Figure 10D), although this is almost entirely due to the differential water retention around M7 previously noted (Figures 7B and 8). We also mapped the residues back to the hexamer tiles, noting that only Y41 is near the pore bottleneck and might impact critical dynamics in this region (Figure S7). The water retention and SASA ratio of Y41 are around 1, both in simulation and experiment, which strongly suggests that the environment around Y41 is unaffected by the change in environment between isolated tiles and assembled shells. As mentioned previously, M7, M16, and M23 are buried in the secondary structure, and Y34 is exposed to the surface but still away from the narrowest part of the pore. K54, P77, P79, and P88 are at the interface between hexamer tiles. Although some of these residues have differences in the water retention and SASA ratio, these residues are likely not actively engaged in pore dynamics or structure.

## Discussion and Conclusions

From the outset, the primary question we were seeking to answer was if future calculations aimed at determining permeability at the molecular scale could assume that the pores are similar, irrespective of their local environment within either shell or a tile within a larger sheet. The direct evidence indicates that this is a reasonable assumption, with Figure 6 showing little difference in the pore diameter, regardless of whether the hexamer and trimer tiles are arranged as they would in the HO shell, or if they are instead tiled into a planar sheet. Given the substantial reduction in system size that a planar sheet-like arrangement provides, we anticipate using these results as the foundation for the computational simplification of determining permeability through the individual pores at the center of the abundant trimer and hexamer tiles found in many BMC shells.

Indeed, the depth at which we had to look to find any differences between shells and sheets is quite remarkable. The water contacts are the same (Figure 7A), and while we do not show it, the SASA analysis is also highly similar between sheets and shells when one looks at monomers without a solvent-exposed edge. The only difference we find is that there are sporadic trapped waters whose lifetimes are a bit longer in the sheet rather than in the shell (Figure 7B). Admittedly, the number of trapped water molecules that contribute to these long lifetimes is not large compared with the total number of water molecules that interact with BMC components. However, since we have multiple copies of the hexamer within our system and can average over 18 monomers where waters may be trapped when conducting our analysis, we are confident that the effect is real.

Our confidence is increased by comparisons to XFMS data for isolated hexamer tiles relative to hexamers within an assembled shell (Table 1). Inferring as we do in Figure S2 that our shell fragment simulation has components that are in similar environments to both experimental systems, with a solvent-exposed edge and a buried edge to individual hexamers, the SASA correlates with XFMS data (Figure 10), analogous to prior results in other systems.^60^ However, for buried residues such as M7, M16, and M23, SASA no longer corresponds to XFMS data, as the SASA is uniformly zero. In this case, water retention times, which more directly measure nanoscale interactions with water, yield better correlations (Figure 10). In particular, the correlation between long-lived water molecules near buried sites such as M7 and the change in the rate of hydroxylation is far stronger than we had anticipated. Initially, the M7 result was thought to be an outlier, but only by using molecular simulation to visualize trapped water molecules can we develop a rational basis for this result.

Zooming out, we think it is helpful to analogize how these BMC protein pores compare with those of typical membrane channels and transporters. Membrane transporters often must go through a conformational change to fulfill their function.^61^ While we do see pore dynamics over our simulation, with the bottleneck radius increasing and decreasing over time (Animations S3 and S4), we find that these dynamics are primarily driven by side chain rearrangements, such as what is highlighted in Figure S4, rather than large scale conformational change as might occur in a membrane transporter. Metabolite-driven gating has been postulated for other trimer pores^24,62,63^ and may well be what occurs in HO shells as well.

Thus, the closest membrane protein analogy for these BMC shell components appears to be that of a channel. Despite starting from a closed state, the trimer opens spontaneously during our simulations, suggesting that the trimer can be gated depending on conditions, analogous to gated ion channels. This was also considered as an explanation for the two particle classes observed cryo-EM studies of the synthetic HO shell.^22^ The hexameric assembly has a smaller variation in pore diameter and is more analogous to a constitutively open channel, such as some aquaporins.^64,65^

While the border between channels and transporters is often murky, channels typically have higher conductances,^66^ which would benefit reactant and product exchange across the BMC shell. With the membrane channel analogy in mind, we anticipate that many small molecules may transit through the central pores with a high permeability. The limiting factor will be molecular size, as we anticipate that sufficiently large molecules will be unable to transit the pore through these tiled BMC shell proteins arrangements. Now armed with a computationally efficient planar arrangement of BMC shell components, we are well positioned to test the high permeability hypothesis explicitly. When these products are tested over multiple metabolic pathways featuring different substrates and products, we hope to develop general rules for transport across BMC shells.

## Data Availability

All input scripts to build and run molecular simulations are made publicly available on Zenodo, together with selected outputs and analysis.^34^

## Supporting Information Available

Molecular dynamics simulation for the sheet system featuring hexameric and trimeric BMC shell tiles (Animation S1) (MP4)
Molecular dynamics simulation of shell fragment from a larger BMC (Animation S2) (MP4)
Animation depicting pore dynamics in BMC trimer and hexamer pores when taken from a shell fragment (Animation S3) (MP4)
Animation depicting pore dynamics in BMC trimer and hexamer pores when taken from a planar shell sheet (Animation S4) (MP4)
Reduced simulation system consisting of three hexameric units and one trimer dimer which can mimic the planar sheet conformation and keeps the system periodic in a conventional orthogonal unit cell (Figure S1); exposed and buried hexamer monomers from our shell fragment simulation (Figure S2); relative distribution of pore radii along the central pore for hexameric and trimeric proteins tiles in either a shell fragment or sheet morphology (Figure S3); top view for open and closed hexamers and stacked trimer observed during the simulation (Figure S4); partially closed conformation predicted by hole program and molecular view of the partially closed conformation of the trimer in sheet conformation (Figure S5); water contacts mapped to each residue of the shell fragment and sheet assemblies (Figure S6); and residue from XFMS analysis mapped onto the hexamer tiles (Figure S7) (PDF)
